# Contralateral exploration and repair of occult inguinal hernias during laparoscopic inguinal hernia repair: systematic review and Markov decision process

**DOI:** 10.1093/bjsopen/zraa020

**Published:** 2020-12-23

**Authors:** N H Dhanani, O A Olavarria, S Wootton, M Petsalis, N B Lyons, T C Ko, L S Kao, M K Liang

**Affiliations:** Department of Surgery, Lyndon B. Johnson General Hospital, McGovern Medical School at UTHealth, Houston, Texas, USA; Department of Surgery, Lyndon B. Johnson General Hospital, McGovern Medical School at UTHealth, Houston, Texas, USA; Department of Pediatrics, Memorial Hermann Children’s Hospital, McGovern Medical School at UTHealth, Houston, Texas, USA; Department of Surgery, Lyndon B. Johnson General Hospital, McGovern Medical School at UTHealth, Houston, Texas, USA; Department of Surgery, Lyndon B. Johnson General Hospital, McGovern Medical School at UTHealth, Houston, Texas, USA; Department of Surgery, Lyndon B. Johnson General Hospital, McGovern Medical School at UTHealth, Houston, Texas, USA; Department of Surgery, Lyndon B. Johnson General Hospital, McGovern Medical School at UTHealth, Houston, Texas, USA; Department of Surgery, Lyndon B. Johnson General Hospital, McGovern Medical School at UTHealth, Houston, Texas, USA

## Abstract

**Background:**

Contralateral clinically occult hernias are frequently noted at the time of laparoscopic unilateral inguinal hernia repair. There is no consensus on the role of contralateral exploration and repair. This systematic review assessed the safety and efficacy of operative repair of occult contralateral inguinal hernias found during unilateral repair.

**Methods:**

PubMed, Embase, and the Cochrane Central Register of Controlled Trials were searched from inception to February 2020. Adults diagnosed with a unilateral inguinal hernia undergoing laparoscopic repair were included. The primary outcome was the incidence of occult contralateral hernias. Summative outcomes of operative and expectant management were reported along with development of a Markov decision process.

**Results:**

Thirteen studies (1 randomized trial, 12 observational cohorts) with 5000 patients were included. The incidence of occult contralateral inguinal hernias was 14.6 (range 7.3–50.1) per cent. Among patients who underwent repair, 10.5 (4.3–17.0) per cent experienced a postoperative complication. Of patients managed expectantly, 29 per cent later required elective repair for symptoms. Mean follow-up was 36 (range 2–218) months. Using a Markov decision process, it was calculated that, for every 1000 patients undergoing unilateral inguinal hernia repair, contralateral exploration would identify 150 patients with an occult hernia. Repair would result in 15 patients developing a postoperative complication and 105 undergoing unnecessary repair. Alternatively, expectant management would result in 45 patients requiring subsequent repair.

**Conclusion:**

Contralateral repair is not warranted in patients with occult hernias diagnosed at the time of elective hernia repair. The evidence is largely based on observational studies at high risk of bias.

## Introduction

Inguinal hernias are common, comprising 75 per cent of all abdominal wall hernias^1^. More than 20 million hernias are estimated to be repaired each year worldwide^1^ and over 800 000 inguinal herniorrhaphies are performed annually in the USA^2^.

Randomized controlled trials (RCTs)^3–5^ have demonstrated that patients undergoing laparoscopic repair of inguinal hernias experience less postoperative pain, have a faster recovery, and have similar recurrence rates to those undergoing open repair. International guidelines^6^ in 2018 stated that either open or laparoscopic repair is recommended for unilateral inguinal hernias, whereas laparoscopic repair is recommended for repair of primary bilateral inguinal hernias.

A potential benefit of laparoscopic repair includes the ability to explore and diagnose the contralateral groin for a clinically occult hernia. Although high rates of occult contralateral hernias have been reported (up to 50 per cent)^7^, the true incidence is unknown. Once an occult hernia has been diagnosed, the optimal management strategy remains unclear. Failure to repair an occult hernia that later becomes symptomatic affects patient well-being, carries the risks of morbidity from a second operation, and places a further burden on the healthcare system. On the other hand, repairing an occult inguinal hernia could be unnecessary in a patient who may never develop symptoms, simply exposing the patient to complications from repair. There is no clear consensus on the role of contralateral exploration and potential repair at the time of unilateral inguinal hernia repair.

The primary purpose of this systematic review was to determine the incidence of occult contralateral hernias diagnosed during laparoscopic unilateral inguinal hernia repair in order to develop a decision analysis model to compare the outcomes of expectant management *versus* concurrent repair.

## Methods

A review of PubMed, Embase, and the Cochrane Central Register of Controlled Trials was performed in accordance with the PRISMA guidelines^8^. ClinicalTrials.gov was searched for ongoing trials. The search included articles published up to February 2020. Search terms included (‘laparoscopic’ or ‘laparoscopy’), and (‘sonography’ or ‘sonographies’ or ‘radiology’ or ‘radiologic’ or ‘CT’ or ‘MRI’), and (‘occult’ or ‘hidden’ or ‘incidental’) and (‘groin’ or ‘inguinal’) and (‘hernia’). No limits or filters (such as study language, study design) were employed. Exclusion criteria were: non-clinical studies, non-human studies, paediatric studies, systematic reviews, meta-analyses, letters, editorials, and/or commentaries. Reference lists of selected articles, systematic reviews, and meta-analyses were reviewed for further articles.

Two authors independently reviewed titles, abstracts, and full-text articles to identify eligible studies. Any discrepancies were discussed and resolved with the principal author.

The study design, definition of occult hernia, number of patients, sex, age, BMI, diagnostic modality, operative technique, incidence of occult hernia, duration of operation, postoperative length of follow-up, early and late complications, and patient-reported outcomes were extracted from each study. The definition of occult hernia as used by the World Guidelines for Groin Hernia Management (WHS) was also obtained^9^. For the purpose of this review, occult hernia was defined using each author’s provided definition.

The risk of bias for each non-randomized study was assessed using the Newcastle–Ottawa Scale^10^.

When possible, treatment effects were pooled. Number needed to treat (NNT) or harm (NNH), defined as 1 – absolute risk reduction, were calculated. Subgroup analysis, defined *a priori*, was performed for total extraperitoneal (TEP) *versus* transabdominal preperitoneal (TAPP) repairs. Comparisons among groups were made using the χ^2^ test.

A Markov decision process was created using a stochastic framework^11^. Specifically, at each decision point, the surgeon chooses an optimal management strategy based on the patient’s observed state, which initially is defined as clinical diagnosis of a unilateral inguinal hernia, then later as the presence or absence of an occult hernia. Treatment modalities were categorized as immediate repair with high risk and high reward; and expectant management, with lower risk and lower reward. Sampling error was calculated using the study population provided in this review compared with the general population undergoing unilateral hernia repair^2^; a sample proportion of 50 per cent and confidence interval of 95 per cent were used.

## Results

**Fig. 1:**
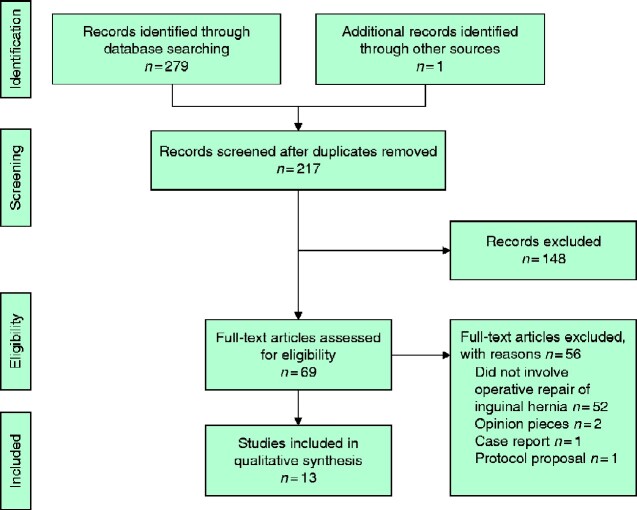
PRISMA flow diagram showing selection of articles for review

A total of 279 studies were identified by the search, with one additional article found through reference review (*Fig. 1*). After removal of duplicates, 217 abstracts were reviewed. A further 148 articles were excluded on the basis of the specified exclusion criteria. After screening, 13 full-text articles were reviewed: one RCT^12^ and 12 observational cohort studies^7^^,^^13–23^.

All included studies were at risk of bias. The observational studies were at higher risk given the lack of blinding, presence of confounders, and selection bias (*Table 1*). The single RCT^12^ benefited from random allocation and blinding of outcome assessors; however, it was unclear whether patients were blinded to the treatment allocation.

**Table 1:** Newcastle–Ottawa Scale assessment of quality of included non-randomized studies

	Selection				Comparability	Outcome		
Reference	Representativeness of exposed cohort	Selection of non-exposed cohort	Ascertainment of exposure	Demonstration outcome of interest was not present at start of study	Comparability of cohorts on basis of design or analysis	Assessment of outcome	Was follow-up long enough for outcomes to occur?	Adequacy of follow-up of cohorts
Bochkarev *et al.*^18^	★	★	★	★	–	★	★	★
Crawford *et al*.^7^	★	★	★	★	★★	★	–	–
Griffin *et al.*^20^	★	★	★	★	–	★	–	–
Imai *et al.*^15^	★	★	★	★	★★	★	★	★
Jarrard *et al.*^16^	★	★	★	★	–	★	–	–
Novitsky *et al.*^21^	★	★	★	★	–	★	–	–
Saggar *et al.*^22^	★	★	★	★	–	★	★	★
van den Heuvel *et al.*^14^	★	★	★	★	–	★	★	★
Chiang *et al.*^23^	★	★	★	★	★★	★	★	★
Lal *et al.*^17^	★	★	★	★	★★	★	★	★
Malouf *et al.*^13^	★	★	★	★	★★	★	★	–
Wu *et al.*^19^	★	★	★	★	★★	★	★	★

The maximum score was ★ for all categories except comparability, for which the maximum score was ★★.

The majority of patients were men (92.6 per cent); one-third of the studies were American and the remainder from either Europe or Asia. Patient BMI ranged from 22.9 to 26.5 kg/m^2^. All studies relied on clinical examination for diagnosis; one study^13^ routinely used ultrasound imaging as a supplement (*Table 2*).

**Table 2:** Study demographics and outcomes

Reference Country	Study design	n	Men	Age (years)	BMI (kg/m2)	Diagnostic modality	Operative repair technique	Incidence of occult hernia (%)	Follow-up (months)	Postoperative complications (%)
Bochkarev *et al.*^18^ USA	Cohort	100	100 (100)	48*	–	Clinical examination	TEP	22.0	24 (4–46)*	17.0
Crawford *et al.*^7^ USA	Cohort	262	244 (93.1)	52^†^	–	Clinical examination	TEP/TAPP	50.1	–	–
Griffin *et al.*^20^ UK	Cohort	306	279 (91.2)	59*	–	Clinical examination	TAPP	22.0	1.5^†^	–
Imai *et al.*^15^ Japan	Cohort	693	523 (75.5)	Open: 68* TAPP/TEP: 70*	–	Clinical examination	Open, TAPP/TEP	15.1	Open: 36 (2–120)* TAPP/TEP: 48 (10–107)*	4.3
Jarrard *et al.*^16^ USA	Cohort	297	–	60.2^†^	26.5^†^	Clinical examination	TAPP	15.8	–	–
Novitsky *et al.*^21^ USA	Cohort	262	244 (92.8)	47.9^†^	26.4^†^	Clinical examination	TAPP	7.3	–	–
Saggar *et al.*^22^ India	Cohort	634	634 (100)	44.4^†^	–	Clinical examination	TEP	8.0	38 (10–82)*	–
Van den Heuvel *et al.*^14^ Netherlands	Cohort	1681	1630 (97.0)	58^†^	–	Clinical examination	TAPP	13.0	112 (16–218)^†^	–
Chiang *et al.*^23^ Taiwan	Cohort	305	262 (85.9)	61.5^†^	23.6^†^	–	TEP	–	30^†^	–
Lal *et al.*^17^ India	Cohort	75	–	37.2^†^	–	Clinical examination	TEP	17	66 (60–72)^†^	–
Malouf *et al.*^13^ Australia	Cohort	234	234 (100)	–	–	Clinical examination; ultrasound imaging for unilateral inguinal hernias	TEP	34.6	1.5^†^	13.0
Thumbe and Evans^12^	RCT	37	37 (100)	Intervention: 53.5* Control: 52*	–	Clinical examination	TAPP	–	Intervention: 12* Control: 15*	–
Wu *et al.*^19^ Taiwan	Cohort	114	98 (86.0)	Exploration: 50.1^†^ No exploration: 56.8^†^	22.9–23.5^†^	–	TEP	33.8	34 (6–66)^†^	5.0

Values in parentheses are percentages unless indicated otherwise; values are *median and ^†^mean, with range in parentheses where available. TEP, totally extraperitoneal; TAPP, transabdominal preperitoneal.

Only five articles^12^^,^^14–17^ explicitly defined an occult hernia (*Table 3*). Definitions provided varied but appeared to be contingent on operative exploration. The WHS^8^ defined occult hernia as ‘an asymptomatic hernia not detectable by physical examination’. The included RCT^12^ used the term ‘incidental’ rather than occult. One study^14^ included both occult and incipient hernias, and defined an incipient hernia as a ‘beginning or looming inguinal hernia’. Occult, incidental, and incipient hernias were pooled in recording outcomes.

**Table 3:** Definition of occult hernia by study

Reference	Definition
**Occult hernia**	
Imai *et al.*^15^	Asymptomatic hernia not detected by physical examination
Jarrard *et al.*^16^	Hernias not identified on physical examination
van den Heuvel *et al.*^14^	Presence of an evident inguinal hernia on laparoscopy
Lal *et al.*^17^	Intraoperative finding of peritoneal protrusion seen traversing beyond the deep ring into the inguinal canal (indirect) or a peritoneal protrusion seen going beyond a visible defect in the fascia transversalis, at a site different from the one diagnosed by preoperative clinical examination
**Incidental hernia**	
Thumbe and Evans^12^	Unsuspected hernia defect without clinically demonstrable hernia
**Incipient hernia**	
van den Heuvel *et al.*^14^	Beginning or looming inguinal hernia; discrete protrusion or bulging of peritoneum is seen but too small and shallow to be regarded as a hernia sac

The incidence of occult hernias was available in 11 studies^7^^,^^13–22^. The cumulative incidence in these studies was 14.6 (range 7.3–50.1) per cent (*Table 2*). Subgroup analysis of the incidence of occult hernias in TEP as opposed to TAPP exploration showed a higher incidence in TEP exploration (21.4 *versus* 13.5 per cent; *P* < 0.001).

**Fig. 2:**
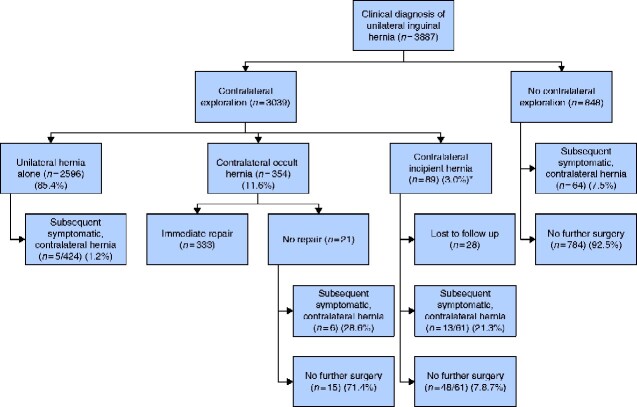
Flow diagram of included study patients *Not repaired at time of initial operation. Occult: hernia not palpable on physical examination. incipient: beginning or looming inguinal hernia—discrete protrusion or bulging of peritoneum is seen but too small and shallow to be regarded as a hernia sac.

In one study^12^, the authors identified but did not repair contralateral occult inguinal hernias. Eventually, 29 per cent of these patients became symptomatic requiring repair (NNT 3.5) (*Fig. 2*), with a mean follow-up of 8 months.

One study^14^ reported the incidence of progression (development of symptoms) among patients with an incipient contralateral inguinal hernia that was not repaired immediately. In this study, 21 per cent of patients eventually developed a contralateral, symptomatic hernia (NNT 5). (*Fig. 2*). The mean interval between initial repair and development of a contralateral, symptomatic hernia was 88 (range 24–210) months. Incipient hernias were classified as occult hernias in analysis of outcomes.

Three studies^15^^,^^19^^,^^23^ reported the incidence of metachronous inguinal hernias, defined as a hernia that developed on the opposite side from the initial hernia repair; 7.5 per cent of these patients required a subsequent operation (NNT 13), with a mean follow-up of 30 months (*Fig. 2*). None of these studies employed contralateral exploration at the time of initial repair.

Two studies^19^^,^^22^ reported the incidence of patients found to have no hernia on contralateral exploration at the time of initial repair who then developed a hernia, totalling five of 424 (1.2 per cent), with a median follow-up of 38 (range 10–82) months.

Four studies^13^^,^^15^^,^^18^^,^^19^ reported complications related to contralateral exploration and/or repair. The incidence of postoperative complications in patients undergoing contralateral exploration was 10.5 (range 4.3–17.0) per cent (NNH 10). Complications included acute pain or discomfort lasting less than 6 weeks (13 per cent), seroma (8.9 per cent), haematoma (5 per cent), and surgical-site infection (1.6 per cent). A small number of patients had acute urinary retention attributed to bilateral repair (1 per cent). Chronic pain, defined as pain requiring oral analgesics for at least 6 months, was reported in two studies^18^^,^^19^, with a total incidence of 2.4 per cent in patients undergoing contralateral exploration and/or repair at follow-up of more than 24 months. Subgroup analysis comparing postoperative complications after TEP *versus* TAPP contralateral repair was not possible as none of the studies that used TAPP repair reported postoperative complications.

One study^13^ presented patient-reported outcomes after contralateral repair *versus* ipsilateral repair alone. Pain and quality-of-life scores were lower at 2 weeks in those undergoing contralateral repair, but no difference was seen at 6 weeks.

One study^19^, involving TEP repair, reported the operative time for contralateral exploration versus no exploration (62 versus 55 minutes); 23 of the 68 patients who had contralateral exploration (34 per cent) had an occult hernia that was repaired immediately. Four studies^17^^,^^18^^,^^20^^,^^23^ reported the operative time for bilateral *versus* unilateral hernia repair, with mean difference of 21.3 min (*Table 4*).

**Table 4:** Duration of operation in included studies

Reference	Duration of operation (min)			
	No exploration	Exploration^§^	Unilateral repair	Bilateral repair
Wu *et al.*^19^	55*	62*	–	–
Bochkarev *et al.*^18^	–	–	38.7 (18–125)^†^	53.9 (35–167)^†^
Griffin *et al.*^20^	–	–	57.5 (24–114)^†^	81.1 (40–142)^†^
Chiang *et al.*^23^	–	–	59.8(29)^‡^	85.2(33)^‡^
Lal *et al.*^17^	–	–	66.2(12)^‡^	87.2(11)^‡^

Values are *median, ^†^mean (range) and ^‡^mean(s.d.). ^§^23 of 68 patients (34 per cent) had an occult contralateral inguinal hernia detected and repaired immediately.

**Fig. 3:**
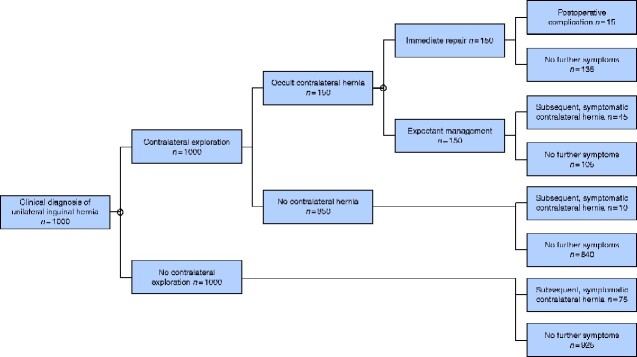
Markov model demonstrating the prognosis of a patient subsequent to the choice of a management strategy Decision points are indicated by diamonds. Sampling error +/– 10 per cent.

A Markov decision process model was created using these outcomes based on numerical simulation of 1000 patients with a clinically diagnosed unilateral inguinal hernia (*Fig. 3*). The first decision point was whether or not to perform contralateral exploration, then proceeded to the patients’ observed state of either having an occult contralateral hernia (15 per cent) *versus* no contralateral hernia (85 per cent). The model then balanced risk *versus* reward in whether or not to repair the hernia immediately using a postoperative complication rate of 10 per cent. The model indicated that, for every 1000 patients undergoing unilateral inguinal hernia repair, contralateral exploration would identify 150 patients with an occult hernia. Immediate repair would result in 15 patients developing a postoperative complication and 105 undergoing an unnecessary repair, whereas expectant management would result in 45 patients requiring subsequent repair.

## Discussion

In this systematic review, the incidence of occult inguinal hernias diagnosed at the time of laparoscopic inguinal hernia repair was 14.6 per cent. Based on the pooled results, when undergoing occult hernia repair, 71 per cent of patients would undergo an unnecessary repair and 10.5 per cent would experience a complication. Alternatively, if the hernia was left unrepaired, less than one-third of patients with an occult inguinal hernia diagnosed during surgery would eventually require a second operation. Therefore, only around 5 per cent of all patients undergoing a unilateral inguinal hernia repair would benefit from contralateral exploration. On average, exploration for, and repair of, occult hernias at the time of unilateral repair does not appear to be warranted. Exploration may be reasonable for symptoms in the absence of physical signs as part of shared decision-making with the patient.

The present study disclosed the absence of a widely accepted definition of occult hernia. Among 13 studies, there were five different definitions and introduction of three new terms: incidental, incipient, and metachronous. In this review, all definitions of occult hernia included lack of a hernia on clinical examination. There is no agreement regarding whether absence of symptoms should also be considered necessary for the diagnosis. Inclusion of this criterion, although clinically meaningful, poses a diagnostic dilemma for symptomatic patients without a palpable hernia. The next step should be to gather a consensus definition for hernias only noted at the time of operation, or by preoperative imaging, accepting that there is substantial disagreement even among radiologists reviewing imaging for the diagnosis of a hernia^24^. Another factor affecting diagnostic certainty is the surgical approach. The included studies reported both TEP and TAPP repairs. TEP exploration may require additional dissection that increases risk of harm (such as epigastric artery/vein or spermatic cord injury), potentially induces iatrogenic weakness, and can make subsequent operations more difficult. Limitations of TAPP exploration include an inability to diagnose small defects and distinguish true cord lipomas from extensions of preperitoneal fat. This may explain the higher incidence of occult hernias with TEP repair in the present review.

Most postoperative complications reported in this review were minor and resolved within 6 weeks; late complications (lasting for 6 weeks or more) included chronic pain and was seen in less than 3 per cent of patients. In previous studies, the incidence of hernia recurrence and chronic pain following laparoscopic inguinal hernia repair was reported to be 0–10 per cent^25^ and 6–15 per cent respectively^26^^,^^27^. It is unknown whether these numbers can be translated to repair of occult hernias, but it seems reasonable to assume that repair of occult hernias would be associated with some hernia recurrence and risk of chronic pain, particularly as bilateral repair has been demonstrated to have worse postoperative complication and reoperation rates than unilateral repair^28^^,^^29^.

In the single RCT^12^ included in this review, 29 per cent of patients with an occult hernia eventually required surgery with mean follow-up of 8 months (NNT 3.5). In an RCT^30^ that evaluated expectant management *versus* immediate open repair in men with an asymptomatic clinically apparent inguinal hernia, 23 per cent crossed over from expectant management to repair over 2–4.5 years (NNT 4.3). The complication rate for those undergoing surgical repair was 21.7 per cent (NNH 4.6).

This review has several limitations. Substantial heterogeneity existed regarding the definition of an occult hernia^12^^,^^14–17^, and so outcomes are subject to bias. Few studies used radiography^13^. Although not adopted widely, some studies^25^^,^^31^ have demonstrated greater sensitivity in diagnosis, albeit at the risk of false positives and additional cost. Most studies in this review were observational, with substantial selection bias and lack of blinding. Duration of follow-up was variable; long-term follow-up of patients diagnosed with an occult hernia that is not repaired is needed to determine the true rate of patients who will eventually become symptomatic. Finally, although men have a greater prevalence of inguinal hernias, women were under-represented in this review. Future studies should report subgroup analyses for women.

Despite low-quality evidence and a substantial risk of bias in the included studies, immediate repair of occult contralateral inguinal hernias diagnosed at the time of elective hernia repair is not justified. Following intraoperative diagnosis of an occult contralateral hernia, more than 70 per cent of these patients will not require treatment. Without contralateral exploration, less than 10 per cent are likely to present for contralateral repair. Immediate diagnosis and repair will result in more complications than expectant management.

## Funding

This research did not receive any specific grant from funding agencies in the public, commercial, or not-for-profit sectors.
